# Mechanisms of cooperation in the plants-arbuscular mycorrhizal fungi-bacteria continuum

**DOI:** 10.1093/ismejo/wraf023

**Published:** 2025-02-08

**Authors:** Shilong Duan, Zexing Jin, Lin Zhang, Stéphane Declerck

**Affiliations:** Université catholique de Louvain, Earth and Life Institute, Applied microbiology, Mycology, Croix du sud 2, bte L7.05.06, Louvain-la-Neuve B-1348, Belgium; State Key Laboratory of Nutrient Use and Management; College of Resources and Environmental Sciences; Key Laboratory of Plant-Soil Interactions, Ministry of Education, China Agricultural University, Beijing 100193, China; State Key Laboratory of Nutrient Use and Management; College of Resources and Environmental Sciences; Key Laboratory of Plant-Soil Interactions, Ministry of Education, China Agricultural University, Beijing 100193, China; State Key Laboratory of Nutrient Use and Management; College of Resources and Environmental Sciences; Key Laboratory of Plant-Soil Interactions, Ministry of Education, China Agricultural University, Beijing 100193, China; Université catholique de Louvain, Earth and Life Institute, Applied microbiology, Mycology, Croix du sud 2, bte L7.05.06, Louvain-la-Neuve B-1348, Belgium

**Keywords:** biological market theory, cross-kingdom cooperation, direct and indirect reciprocity, holobiont, “surplus C” hypothesis

## Abstract

In nature, cooperation is an essential way for species, whether they belong to the same kingdom or to different kingdoms, to overcome the scarcity of resources and improve their fitness. Arbuscular mycorrhizal fungi are symbiotic microorganisms whose origin date back 400 million years. They form symbiotic associations with the vast majority of terrestrial plants, helping them to obtain nutrients from the soil in exchange for carbon. At the more complex level, soil bacteria participate in the symbiosis between arbuscular mycorrhizal fungi and plants: they obtain carbon from the exudation of hyphae connected to the roots and compensate for the limited saprophytic capacity of arbuscular mycorrhizal fungi by mineralizing organic compounds. Therefore, plants, arbuscular mycorrhizal fungi and soil bacteria constitute a continuum that may be accompanied by multiple forms of cooperation. In this review, we first analyzed the functional complementarities and differences between plants and arbuscular mycorrhizal fungi in arbuscular mycorrhizal symbiosis. Secondly, we discussed the resource exchange relationship between plants and arbuscular mycorrhizal fungi from the perspective of biological market theory and “surplus carbon” hypothesis. Finally, on the basis of mechanisms for maintaining cooperation, direct and indirect reciprocity in the hyphosphere, induced by the availability of external resource and species fitness, were examined. Exploring these reciprocal cooperations will provide a better understanding of the intricate ecological relationships between plants, arbuscular mycorrhizal fungi and soil bacteria as well as their evolutionary implications.

## Introduction

Cooperation between species has always been at the heart of ecosystems, with many organisms in nature maintaining a network of interactions and interdependencies [1]. These interactions are often mutually beneficial, but can also be mutually exclusive, playing a crucial role in the physiology and adaptation of organisms to their environment [2]. Soil microorganisms constitute the most biologically diverse community of organisms in terrestrial ecosystems, with tens of millions of bacteria, archaea, fungi, viruses, and microeukaryotes coexisting underground [3]. One gram of surface soil contain more than 10^9^ bacterial and archaeal cells, 200 meters of fungal hyphae, trillions of viruses, and thousands of protists [4]. They engage in a wide variety of social interactions such as predation, parasitism, symbiosis, and mutualism to survive and evolve [5]. This may involve individuals of the same species or individuals of two or more species belonging to the same kingdom (e.g. bacteria versus bacteria) or different kingdoms (e.g. bacteria versus fungi), or even different domains (e.g. eukaryotes versus prokaryotes) [6]. Cross-kingdom cooperation can increase the stability and productivity of ecosystems by bringing phylogenetically distant species closer together. The benefits of cooperation tend to turn into trophism when one party gains access to resources from which the other party is excluded or has only limited access [7].

**Figure 1:**
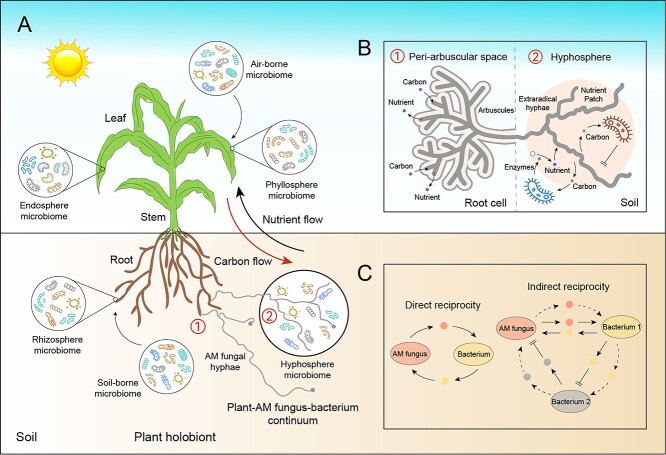
(A) Overview of the plant holobiont and the plant-arbuscular mycorrhizal (AM) fungus-bacterium continuum. Plants host a wide diversity of microorganisms both inside and outside their tissues, in the endosphere and ectosphere (phyllosphere and rhizosphere), respectively. These microorganisms participate in plant health, forming the plant holobiont. AM fungi are an essential member of the holobiont. Their hyphae connect plant roots to soil microbial communities, forming the plant-AM fungus-bacterium continuum accompanied by top-down carbon flow and bottom-up nutrient flow. (B) Two key interfaces in the plant-AM fungi-bacteria continuum. AM fungal intraradical hyphae grow into the intercellular spaces of root cortical tissues and form highly branched arbuscules, thereby constituting the peri-arbuscular space interface for AM fungi to facilitate the exchange of C and nutrients with plants. In parallel, AM fungal extraradical hyphae grow into soil crevices and micropores, providing a habitat for soil bacteria thanks to their vast network of hyphae. The hyphosphere becomes an interface for AM fungi to facilitate the exchange of C and nutrients with soil bacteria. Numbers represent the location of these two magnified interfaces in the plant-AM fungus-bacterium continuum. (C) Direct and indirect reciprocity in the hyphosphere. Direct reciprocity means cooperation through metabolic exchange or provision of available resources between AM fungus and bacterium at a lower cost for both participants. Indirect reciprocity typically involves a tripartite interaction: (i) AM fungus and bacterium 1 directly exchange available resources, whereas the metabolites secreted by bacterium 1 mitigate the potential harm inflicted by bacterium 2 on AM fungus (solid line). This is an indirect reciprocity currently found in the hyphosphere. (ii) AM fungus assists bacterium 1, bacterium 1 subsequently aids bacterium 2, and bacterium 2, in turn, benefits AM fungus (dotted line). This is an indirect reciprocity that needs further verification through experiments.

Plants do not exist in isolation in an ecosystem. A large number of microorganisms inhabit various organs (e.g. leaves and roots) of the plants, forming the unique microbiome, which cooperates closely with the plants and influences their performance and the function of the ecosystem. These outcomes are the fruit of the plants themselves and their associated microbiomes, collectively forming the holobiont (Fig. 1A) [8, 9]. AM fungi are essential members of the holobiont, forming symbiotic associations with more than two thirds of terrestrial plants [10], and engaging in bi-directional exchanges of carbon (C) sources from plants to fungi and of mineral compounds (e.g. phosphorus – P and nitrogen – N) from AM fungi to plants [11]. In this way, plants and AM fungi provide what they can at low cost in exchange for resources that would otherwise be impossible or more difficult to obtain [12]. This mutualistic cooperation can extend to bacterial partners, as the extraradical hyphae of AM fungi are the perfect place for many soil-dwelling microorganisms to grow. This dynamic interface is commonly referred to as the hyphosphere, where the physical, chemical, and biochemical characteristics are different from the bulk soil due to the influence of hyphal exudates. The hyphae of AM fungi thus connect plant roots to soil microbial communities, forming plants-AM fungi-bacteria continuum (Fig. 1B) [13, 14].

Similar to rhizosphere, hyphosphere is a C-rich ecological niche that can, to some extent, break bacterial dormancy and modify the composition of bacterial communities [15]. Although AM fungi can widely explore soil for resource exchange with plants (e.g. minerals for C), their limited saprophytic capacity prevent them from exploiting organic compounds that are omnipresent in the soil [16, 17]. Some bacteria colonizing the hyphosphere could compensate for the lack of AM fungal saprophytic ability by their strong capacity to mineralize organic P and hydrolyze organic N from the soil in exchange for C and energy [18, 19].

Recent studies have suggested the existence of two distinct modes of cooperation, namely direct and indirect reciprocity, between AM fungi and soil bacteria, which are attributed to their metabolite’s exchanges (Table 1) [24, 25]. Metabolites can be released into the environment through active or passive ways. Species may secrete these compounds actively in order to promote cooperation and modify adaptation strategies to their surroundings, or passively, due to an inability to retain these compounds in their cytoplasm as a result of leakage issues or surplus nutrients [26, 27].

**Table 1:** Terms used to describe the relationship between plants, arbuscular mycorrhizal fungi and soil bacteria.

Term	Interpretation	Conceptual basis
Cooperation	Mutually beneficial interaction between two or more individuals. Defined in a strict sense, cooperation is used when these individuals are members of the same species and mutualism is used when they belong to different species or kingdoms.	[1]
Mutualism	Mutually beneficial interaction between members of different species. Conceptually equivalent to cooperation between unrelated members of the same species.	[20]
Continuum	Biological continuum refers to a dynamic system encompassing life activities composed of plants, AM fungi, and hyphosphere bacteria, including top-down C flow and bottom-up nutrient flow.	[13]
Holobiont	Initially, holobiont referred to a simple biological entity consisting of a host and a single inherited symbiont. It was extended to include the host and its associated microbial community (also known as the microbiome, which corresponds to the collection of microorganisms that interact with the host, ranging from mutualism to parasitism).	[21]
Direct reciprocity	Direct reciprocity is based on repeated encounters between two different species, and the two species must be able to provide assistance that is less costly for the donor than beneficial for the recipient. “Individuals will tend to help those who help them”.	[22]
Indirect reciprocity	Indirect reciprocity always involves the appearance of a third party, where the actor may not provide benefits directly to the donor but maintains cooperation by influencing third-party partners. “You help me, I help others” or “I help you, others help me” are the most intuitive form of indirect reciprocity.	[23]

Direct reciprocity means that AM fungi release exudates on the surface of their hyphae which feed specific microbiota, which in turn help the AM fungi to acquire nutrients to improve the fitness of AM symbiosis [18, 28]. However, in the case of direct reciprocal activity, there is a risk of “cheaters”, where certain soil bacteria benefit from the generosity of others without making a personal contribution. Indirect reciprocity allows bacteria who dominate the cooperation (i.e. “cooperators”) to interfere or suppress the cheaters to gain access to resources or harm themselves, thereby safeguarding their interests and fitness [24, 25]. Therefore, the direct and indirect reciprocity are two mechanisms that maintain cooperation between unrelated species in the hyphosphere (Fig. 1C).

In this review, we first explored the functional complementarities and differences in the symbiosis established between AM fungi and plants. Secondly, the resource exchange relationships between AM fungi and plants based on biological market theory and “surplus C” hypothesis were analyzed. Finally, we examined the current knowledge about direct and indirect reciprocal cooperation between AM fungi and soil bacteria in the hyphosphere, which is driven by species fitness and resource availability. These mechanisms are major drivers of biological interactions in the subsurface, of long-standing interest to plant and microbial ecologists.

## Exploring the cooperative mechanism of AM symbiosis: evolutionary history, biological market theory and the “surplus C” hypothesis

Over the course of evolution, AM fungi have refined their biotrophic abilities, using their hosts as a source of C and as protective niches, whereas plants have developed multiple strategies for adapting symbionts to cope with resource scarcity and environmental changes [29, 30]. AM symbiosis establish a “division of labor” or “resource division network”, which can even extend to soil bacteria: plants secrete C fixed by photosynthesis which feeds their AM fungal partners, and AM fungi and their associated bacteria, transfer mineral nutrients to plants in order to obtain C for their metabolism (Fig. 1) [13]. The mechanisms of C and mineral nutrient flow in the AM symbiosis have been extensively scrutinized and validated using genetic, molecular, and cellular tools [13, 31]. Here, we focused mainly on evolutionary history, biological market theory, and the surplus C hypothesis to study the transfer of resources in the symbiosis between plants and AM fungi and their stable existence in the ecosystem. This will lead to a better understanding of the reciprocal cooperation between AM fungi and soil bacteria in the hyphosphere.

### Evolution is the origin of functional complementarity and differentiation in AM symbiosis

It is widely accepted that the formation of symbiosis between plants and soil fungi has been instrumental in the transition of plants from aquatic to terrestrial environments [32, 33]. This hypothesis is supported by original descriptions from fossil records [34, 35], paleobotanical data [36, 37], and phylogenetic analyzes based on fungal DNA sequences [38, 39]. In the early studies of symbiotic functions, the assistance provided to the host in acquiring P was established as the most emblematic feature of AM fungi. They acquire P beyond the nutrient-depleted zone of rhizosphere to meet plant nutrient needs by accessing soil pores that are inaccessible to roots and by producing an enormous network of extraradical hyphae that extend several cm from the roots [40]. Some fungi even deliver 70%–100% of the overall P obtained by plants [41, 42]. In addition to nutrient acquisition, plants colonized by AM fungi generally show greater tolerance to biotic and abiotic stresses, which is not simply a consequence of better nutritional status [43, 44]. The beneficial properties of AM symbiosis and their positive effects on plant health and fitness have been demonstrated in many species, covering a wide variety of plant lineages, and the perpetuation of AM symbiosis also explains their ubiquitous distribution in the plant kingdom [31, 45]. However, contrary to popular opinion, AM symbiosis is not necessarily beneficial to plant fitness and can even have a negative impact on plant growth - more often than not, the expected effect on yield and mineral nutrition has not been observed [46–48]. This is not surprising, as assessing the impact of AM fungal inoculation on plants is difficult due to the high variability and uncontrollability of AM fungal inoculation in the field. The overall productivity of plants depends on the interaction of various factors including environment, climate, plant genotype, soil type, and agricultural practices [49–51]. The stable growth and development of AM fungi in agricultural fields, combined with the establishment of an efficient symbiotic relationship with plants, is the cornerstone of achieving positive effects. Appropriate farming practices, such as low or no tillage, plant diversification, and organic farming, can encourage the development of native communities of AM fungi, and the introduction of AM fungi can also be an effective strategy for restoring soils that are naturally impoverished and low in AM fungi [52, 53].

Over the past decade, the exploration of AM fungal genomes has provided clues for unveiling key metabolic features of AM symbiosis. Mechanisms of convergent evolution may have shaped the genomes of AM fungi, with their transition from saprophytes to symbionts mainly involving the massive loss of plant cell wall-degrading carbohydrate-active enzymes (CAZymes), a lack of fatty acid synthases, a reduced ability to synthesize secondary metabolites and the co-selection of genes present in the saprophytic ancestor to fulfill the new symbiotic function [16, 54–56]. In saprotrophic fungi, CAZymes play an essential role in the degradation of soil organic litter. They obtain nutrients by decomposing dead or decaying animal and plant residues and other organic matter to maintain normal life activities [57]. However, substantial evidence confirms that AM fungi are unable to reproduce and survive in non-symbiotic conditions [58, 59]. Lipids serve as energy and C building blocks for AM fungal growth, but the genes encoding multidomain cytosolic fatty acid synthase subunits, which are required for fungal de novo fatty acid synthesis, are supposedly absent from the genomes of all AM fungi as observed in *Rhizophagus irregularis*, *Gigaspora margarita*, and *Gigaspora rosea* [55, 60, 61]. Deletion of multiple fatty acid biosynthetic enzyme genes such as *FatM* and *RAM2* in plants severely impairs AM fungal colonization in roots [62, 63], indicating that AM fungi are fatty acid auxotrophs and their growth and development depend on lipids received from the host plant. In addition, the invertase-encoding and thiamine-encoding genes are absent from AM fungal genomes sequenced to date, further supporting their biotrophic status [64]. These functional complementarities and functional differentiations allow the relationship between plants and AM fungi to be preserved in natural selection and evolution, and gradually evolve into the typical mutualistic symbiosis.

### Application of biological market theory in AM symbiosis

The evolution and development of the AM symbiosis is characterized by a transfer and exchange of resources, which is a typical feature of the biological market. Thus, biological market theory provides a conceptual framework for analyzing the cooperative exchange of resources between plants and AM fungi, allowing us to explore the rewards of fitness they obtain by providing resources in changing environments [65–67]. In such market, individuals tend to profit by distinguishing potential trading partners based on the quality and quantity of commodities they offer [68]. In the symbiosis between plants and AM fungi, the commodities exchanged are C provided by the plants and mineral nutrients provided by the AM fungi. These entities do not have a common currency of exchange, which requires the use of an exchange rate between C and mineral nutrients to express the relative value of these commodities, with the partner offering the best exchange rate being the most favored [69, 70]. Biological market theory provides a balanced view of partner selection, based on different individual perspectives, rather than adopting a traditional plant-centric model [71, 72]. For both plants and AM fungi, the costs and benefits directly affect their fitness. If the benefits outweigh the costs, the individual fitness increases, and if several individuals in a population experience the same balance between costs and benefits, the population grow [12]. In addition, biological market theory allows individuals to manipulate or influence exchange rates when the availability of resources fluctuates, and to make judicious choices about whether to enter into cooperative relationships with other individuals. The actual price for a given exchange reflects a combination of various dynamic factors, including supply and demand as well as nutrient acquisition efficiencies of partners [46, 67].

Both plants and AM fungi have been shown to be able to exploit changes in available resources to impose favorable trading conditions, and higher returns encourage them to invest more in exchanges [11]. For example, plant nutritional and physiological status largely influence C allocation. Under P deficient conditions, host plants can activate a series of adaptive responses regulated by phosphate starvation response (PHR) proteins, including key genes for AM fungi to colonize the roots and exchange nutrient from plants. Conversely, high P condition strongly inhibits the movement of PHR proteins towards the nucleus and/or their binding to the promotors of targeted genes, which hinders root colonization by AM fungi and reduces C allocation [73]. For AM fungi, their P trading strategies with plants in the extraradical hyphal networks are not uniform. The heterogeneity and drastic changes of P resource in the environment can exacerbate the flow of P from hyphae to the host [71, 72]. AM fungi respond to high variation in P resources by increasing total P distributed to host plants, decreasing allocation to storage in the fungal network, and differentially moving P resources within fungal network from rich to poor patches of nutrients [71]. When available P resources decline sharply, AM fungi compensate for the lack of P resources by moving P within the hyphae closer to host roots. If available P resources increase, they also store excess nutrients until root demands increase to get larger C returns [72]. Although biological market theory is well supported in mycorrhizal research, there are still numerous experiments confirming that the C and P exchange between plants and AM fungi does not adhere to the predictions of this theory, and the partners with relatively low or even no value also exist stably [74–76].

In natural ecosystems, most plants are colonized by multiple AM fungi, and AM fungi are always shared by neighboring plant species. These AM fungi can connect together different plant species, among which some can be chlorophyll-free (i.e. myco-heterotrophic species), therefore obtaining their nutrients from AM fungi, whereas AM fungi themselves receive C compounds from the surrounding autotrophic plants, which is contrary to biological market theory [77–79]. Consequently, greater caution should be exercised when using biological market theory to elucidate mutualism between plants and AM fungi. It is imperative to integrate the specific characteristics of AM symbiosis into evolutionary models, in particular the relationship between the complex hyphal networks and diversity of plant root traits, the strength of AM fungal sinks and plant sources, the residual cargo exchange, and environmental conditions for AM symbiont growth [80, 81].

### Application of “surplus C” hypothesis in AM symbiosis

Unlike the biological market theory, which assumes that plants actively identify fungal partners and adapt their C allocation strategies accordingly, the surplus C hypothesis proposes that C transfer by plants may be the consequence of a surplus of C produced by photosynthesis [67, 82]. The surplus C hypothesis is based on the source-sink dynamics of plants and avoids the question of the existence of myco-heterotrophic species, which is difficult to interpret in the framework of biological market theory [79]. It refers to the process of transferring C from tissues or organs of relatively high content or concentration to those of low, irrespective of the underlying mechanism governing C transfer [83]. Consequently, in the presence of a surplus of C in the leaves or the whole plant, they may not actively exchange it with AM fungi for mineral nutrients, opting instead to discharge C. This imposes no cost on the host plants, has no potential harmful effects on their growth or other functions, and does not even require active regulation [82, 84]. The amount of C transferred to AM fungi depends on the plant’s metabolic processes and the strength of the fungal C sink and appears to be independent of the transfer of fungal mineral nutrients [67]. Plant nutrient deficiencies and even growth limitations may not disrupt the process of phloem loading, which allow for the translocation of any surplus photosynthetic products from the source leaves to the sink organs such as branchlets, stems, and roots [85, 86], mitigating the toxicity of photosynthetic C accumulation in leaves.

The surplus C hypothesis does not take into account the form, mechanism, and regulatory processes involved in the C transfer from plants to AM fungi. This may be why the hypothesis is not widely accepted by the research communities. If there is simply a surplus of C, the monosaccharides resulting from sucrose cleavage constitute the primary form of C assimilated by AM fungi, and obviously, lipids serve as the main C source for AM fungi to fulfill their growth and development requirements [63, 87]. De novo biosynthesis of fatty acids occurs in root cells via a set of conserved biochemical reactions. It is an energy-consuming process regulated by the mycorrhizal-specific transcription factors RAM1 and WRI5a [63, 87, 88]. Synthesized fatty acids also necessitate transportation to AM fungi via mycorrhizal-specific fatty acid transporters STR/STR2 situated on the plant plasma membrane [89, 90]. If AM fungi do not provide mineral nutrients or other fitness rewards, they can be a burden for plants. In addition, a large number of studies have demonstrated that the composition of root exudates varies considerably under different nutrient conditions, exerting a profound stimulatory effect on the rhizosphere soil microbial community, which is commonly regarded as the result of plants actively adapting to their environment [91, 92]. For example, in scenarios where the availability of soil P is limited, plants can excrete considerable amounts of carboxylates, especially malic acid and citric acid [93, 94]. The excretion of these compounds may function as a strategy used by plants to acquire P, rather than serving as a means to discharge surplus C.

Overall, the biological market theory serves as an evolutionary framework to elucidate the origin and development of mycorrhizal symbiosis, whereas the surplus C hypothesis exhibits limited association with evolutionary history and primarily emphasizes the intensity of source-sink dynamics [67, 79]. Considering the rationality of the above two perspectives, they need to be balanced in the study of nutrition and ecology of mycorrhizal research.

## Maintaining the cooperation between AM fungi and soil bacteria in the hyphosphere: Direct and indirect reciprocity

Mutualism between plants and AM fungi takes place in the peri-arbuscular space of roots, where intraradical hyphae of AM fungi obtain C to sustain their metabolic activities. Cooperation between AM fungi and bacteria occurs in the hyphosphere, where AM fungi provide C to soil bacteria through their vast hyphal networks. In this plants-AM fungi-bacteria continuum, multiple factors can affect the cooperation and stable coexistence in the complex environment. Plants may establish symbiosis with different AM fungal partners depending on their root traits and photosynthetic efficiency [81]. Alterations in AM fungal communities or AM fungal species lead to different hyphosphere bacterial communities, generally due to the differences in the composition of their exudates and/or by different developmental and metabolic characteristics [95]. The availability of nutrients in the environment influences the AM symbiosis by altering the cooperative strategies between plants and AM fungi, which in turn influences the composition of exudates [96], thereby attracting or repelling soil bacteria, and mediating direct and indirect reciprocity in the hyphosphere. Some beneficial bacteria (e.g. *Rahnella aquatilis* and *Devosia sp.*) can function in synergy with AM fungi to improve plant performance via fine-tuned communication at the hyphosphere and peri-arbuscular interfaces [14, 97]. In contrast, certain bacteria engage in competition with AM fungi for nutrients, thereby reducing the fitness of AM fungi, which may be adversely affected by the antagonism exhibited by other beneficial bacteria [25]. Direct reciprocity and indirect reciprocity are two mechanisms to maintain cooperation between AM fungi and soil bacteria in the hyphosphere (Fig. 1). Here, we discussed these potential mechanisms via a number of study cases.

### Carbon and mineral resource exchange is the driving force behind direct reciprocity

Many studies have shown that AM fungi actively select but do not randomly cooperate with soil bacteria [19, 25] possibly because of their limited exo-enzymatic repertoire. The soil bacteria that rely on exudates released by hyphae for survival often have the ability to mineralize organic P and/or hydrolyze organic N, and their population abundance in the hyphosphere is substantially different from that in the bulk soil [15].

**Figure 2:**
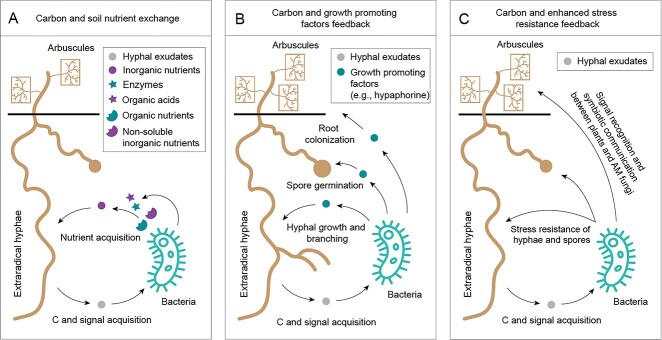
Schematic diagram of direct reciprocity between arbuscular mycorrhizal (AM) fungi and soil bacteria in the hyphosphere. Direct reciprocity based on (A) carbon and soil nutrient exchange, (B) carbon and growth promoting factors feedback and (C) carbon and enhanced stress resistance feedback. AM fungi release exudates to feed soil bacteria, and in return, these bacteria release a variety of enzymes and organic acids, transforming initially inaccessible nutrients into inorganic forms that are easily assimilated by AM fungi. These bacteria can secrete growth promoting factors to stimulate hyphal growth and branching as well as spore germination. In addition, some bacteria stimulated by hyphal exudates can enhance AM fungal resistance and symbiotic communication with host plants.

The exchange of C and mineral nutrients lead to a direct reciprocity between AM fungi and bacteria, a common mode of cooperation in the hyphosphere involving many fungal and bacterial species [28, 95, 98] (Fig. 2A). Experiments conducted in vitro provide conclusive evidence of this reciprocity. Each gram of hyphae (dry weight) of *Rhizophagus irregularis* can release approximately 30 mM of C-containing compounds within four weeks, mainly in the forms of low molecular weight sugars, amino acids, and carboxylates [18, 28]. Different soil bacteria have different C metabolism profiles and may preferentially use different forms of compounds [25]. For example, *Rahnella aquatilis* (a phosphate-solubilizing bacterium) prefers fructose to glucose, whereas *Pseudomonas fluorescens* (a complete denitrifying bacterium) prefers carboxylates [18, 99]. However, if these C-containing compounds are secreted passively (without consuming energy), although this represents surplus C for the AM fungus, similar to the surplus C hypothesis of plants, it does not support the reciprocity cooperation. Intriguingly, the exudates released by the hyphae may not be a purely passive process, but a targeted response that occurs upstream of the passive process of root exudation, which could be controlled by the AM fungi and consume energy [100]. Under different nutritional conditions, the growth of AM fungi can cause significant changes in the composition of their exudates, and even the release of some signaling molecules [96, 101]. Therefore, AM fungi actively provide soil bacteria with C sources needed for growth and stimulate their saprophytic ability, thereby increasing the availability of mineral nutrients to AM fungi. This was well shown with *R. aqualitis* [102] which can swim through the thick water film surrounding the extraradical hyphae of *R. irregularis* towards organic P patches, where it extends the ability of *R. irregularis* to efficiently utilize this otherwise inaccessible P source. AM fungal hyphae were also reported to help the migration of *Sinorhizobium meliloti* to the roots of leguminous plants, thereby triggering nodulation [101]. Specific signals (i.e. flavonoids) are released by the hyphae connected to the legume, serving as chemoattractants. Conversely, *Sinorhizobium meliloti* stimulates the flow of cytoplasm/protoplasm within the hyphae, likely increasing the release of nutrients and signals [101]. In another study [19], it was observed that two chitinolytic *Paenibacillus* sp. isolates that relies on fungal exudates for growth, increased the efficiency of chitin uses as a N source by the extraradical hyphae of *R. irregularis*.

The experimental design and optimization targeting AM fungal and bacterial cooperative mechanisms such as those described above typically consider isolated cells of a single species [19, 97]. Although this reductionist approach aims to simplify the process, it creates a situation that rarely occurs in nature. In the natural environment, microorganisms thrive in complex communities, where the fitness of individual species depends on interactions with other species in the population. In the hyphosphere, a variety of soil bacteria that interact closely with AM fungi can stably exist and form the hyphobiome. At the order level, the bacterial network is dominated by Myxococcales, Betaproteobacteriales, Fibrobacterales, Cytophagales, and Chloroflexales [103, 104]. The core bacterial members are essentially correlated with their phosphatase activity, which can mineralize a large amount of organically bound P in soil [104]. High-throughput stable isotope probing experiments provided further evidence that bacteria colonizing the hyphosphere can lead to an increase in the ability of AM fungi to acquire organic nutrients from the soil, as these beneficial bacteria have a large number of genes encoding carbohydrate-degrading enzymes in their genomes [105]. This is crucial for maintaining the mutualistic symbiosis between AM fungi and plants, as mineral nutrients are the cargo they exchange with plants for C, which determines the growth and development of AM fungi [97]. If AM symbiosis is compromised, extraradical hyphae may experience C limitation, hindering their ability to explore the soil, which affects reciprocal cooperation in the hyphosphere. In addition, some bacteria (e.g. *Paenibacillus validus* and *Pseudomonas monteilii*) dwelling in the hyphosphere can promote spore germination, hyphal growth, and branching by producing growth promoting factors, thereby improving the colonization efficiency of AM fungi within roots (Fig. 2B). These bacteria are also thought to have the ability to enhance AM fungal resistance and facilitate mycorrhizal symbiosis establishment [106] (Fig. 2C). Therefore, the hyphosphere, a critical and active zone of soil, addresses the issue of plant, AM fungal and bacterial resource scarcity and fitness in the ecosystem to a certain extent and promotes their direct reciprocity [28]. However, the internal and external conditions that affect this direct reciprocity are unclear. The extent to which AM fungi provide available C to soil bacteria, rather than passive secretion due to the surplus C, still needs to be further explored.

### “Cooperators” secrete antibiotics to suppress “cheaters” is the driving force behind indirect reciprocity

**Figure 3:**
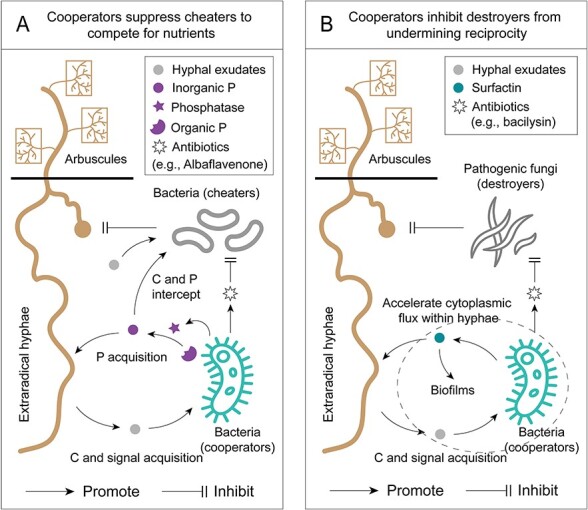
Schematic diagram of indirect reciprocity between arbuscular mycorrhizal (AM) fungi and soil bacteria in the hyphosphere. (A) Bacteria (cooperators) absorb hyphal exudates and produce phosphatases that mineralize soil organic phosphorus (P) into usable inorganic P for AM fungi. Other bacteria (cheaters) do not produce phosphatases but take advantage of inorganic P and hyphal exudates, competing for these valuable nutrients. Cooperators can produce antibiotics (e.g. albaflavenone) to suppress the survival of cheaters and maintain reciprocity [25]. (B) Bacteria (cooperators) absorb hyphal exudates and secrete surfactin to facilitate AM fungi to obtain carbon from host plants, thereby accelerating cytoplasmic flux within hyphae to enhance the growth of AM fungi. Cooperators can produce antibiotics (e.g. bacilysin) to inhibit the fitness of pathogenic fungi (destroyers) to prevent their infestation on AM fungi [24].

Indirect reciprocity in the hyphosphere may be dominated by specific bacterial species, often accompanied by the production of antibiotics or antimicrobial compounds [24, 25]. *Streptomyces* sp. isolated from the hyphosphere can use different forms of hyphal exudates to grow rapidly and plays a decisive role in the early stages of bacterial community formation [25]. *Streptomyces* sp. D1 has the ability to produce phosphatases that are efficient in mineralizing organic P, thereby increasing P availability for AM fungi. In low-P soils, soil bacteria tend to compete with AM fungi for P to prioritize their own resource needs [28]. Therefore, for those bacteria (i.e. the cheaters such as *Pseudomonas* sp. H2 and Paenarthrobacter sp. 31) that contribute little or no to P availability, their acquisition of AM fungi-derived C may not maintain the stability of reciprocal cooperation, and even inhibits the synthesis and transfer of poly-P in the extraradical hyphae of AM fungi. Intriguingly, Streptomyces sp. D1 strongly alters the hyphosphere bacterial communities by inhibiting certain bacteria with low phosphatase efficiency. Conversely, Streptomyces sp. D1 generally does not impact the bacteria with high organic P mobilization capability as these bacteria do not contribute to P resource shortage [25]. Cultured cells and exudates of Streptomyces sp. D1 inhibited by nearly 40% the growth of Pseudomonas sp. H2, by synthesis and secretion of albaflavenone (bactericidal antibiotic). A review of the genome of Streptomyces sp. D1 found that it contains a large number of genes related to antibiotic synthesis, including types I, II, and III polyketide synthases, non-ribosomal peptide synthases, and terpene. The expression of terpenoid backbone biosynthesis-related genes that synthesize albaflavenone precursor substances is generally increased during the interactions in the hyphosphere [25]. These evidences suggest that Streptomyces sp. D1 actively produced antibiotics to punish cheaters in the hyphosphere bacterial community to maintain the stability of mutualistic cooperation between AM fungi and soil bacteria (Fig. 3A). Streptomyces sp. may be a species with cooperative advantages that can limit cheater behavior and negatively affect the fitness advantage of the cheater.

The cheaters frequently undermine the stability of the community by snatching resources and space [27, 107]. In the hyphosphere, the cheating bacteria possess the capability to compete for available C and P, garnering benefits from the resources generated by other collaborators and refraining from contributing to the costs of producing these resources [25]. For the majority of species, their fitness is generally achieved by increasing benefits or reducing costs. If the cheaters proliferate in an uncontrolled manner, they impose a burden on cooperative species, and with the invasion of cheaters, the proportion of resource producers in a population tends to decline to diminish to the brink of population collapse [108, 109]. The true cheating occurs when the relative fitness of the cheater increases above and that of the cooperator decreases below the average fitness of the population, which has been extensively verified in other studies [110–112]. In addition, some soil bacteria (i.e. “destroyers”, such as Burkholderia) have the potential to either feed on hyphae or generate antifungal agents that antagonize AM fungi and suppress the growth of hyphae [113, 114]. However, in complex microbial communities, the question arises as to how to accurately distinguish the identities of cooperators, cheaters, and even destroyers. Their functions and roles as well as the reciprocal mechanisms still need to be explored and verified via more ingenious experimental designs and innovative methods.

Spatial structure is one of the fundamental mechanisms influencing the resilience of cooperation. It allows cooperating species to isolate themselves from cheaters, thus facilitating the formation of indirect reciprocity [115]. Spatial structures at multiple length scales exist in bacterial biofilms, which are multicellular aggregates formed by a matrix composed of biopolymers and proteins. The spatial association within species can benefit themselves, whereas distances between species determines the transport of dispersible resources [116]. The interaction networks within biofilms depend largely on the spatial structure of the biofilms—i.e., the arrangement in space of different microbial species, which affects the resource acquisition and stable survival of bacteria in the ecosystem [115]. If different strains and species mix spatially within the biofilms, bacterial cells interact closely with other species, often in an antagonistic manner, and natural selection tends to favor those species that dominate over competitors [117]. In the hyphosphere, the expression of genes involved in biofilm formation and regulation of *R. aquatilis* is activated by *R. irregularis*, which may play an important role in the early stage of the formation of stable reciprocity [118]. The formation of biofilm aids the proliferation of the species, inhibits the colonization by competitors, and protect scarce resources from cheaters [119]. Very recently, an indirect reciprocity has been reported among *R. irregularis*, *Bacillus velezensis* and mycoparasitic fungi, which is closely related to the formation of biofilm by *B. velezensis* on the hyphae surface of *R. irregularis* [24]. *B. velezensis* stimulated by *R. irregularis* C and signals released at the hyphae surface can release several antimicrobial compounds in its biofilm (e.g. bacilysin, a bioactive secondary metabolite with antimicrobial activity), which can form a chemical barrier protecting the AM fungi from microbial aggressors in order to compensate for the low natural potential of AM fungi to produce antibiotics [24]. This indicates that *B. velezensis* and *R. irregularis* dominate reciprocal cooperation and possess the potential to inhibit destroyers from interfering their stable coexistence (Fig. 3B). In addition, these mutual benefits are also extended to the plant via the provision of enhanced protection against *Botrytis cinerea* via the induction of systemic resistance [24]. Although examples are rare, indirect reciprocity represents a novel form of cross-kingdom cooperation in the hyphosphere, contributing to the stability and development of plant-associated microbial communities in a favorable direction.

## Conclusions

Plants-AM fungi-bacteria continuum represent a model of multi-level cross-kingdom cooperation. Plants, through their intimate association with AM fungi, modulates beneficial direct and indirect reciprocity between AM fungi and soil bacteria in the hyphosphere that improve the adaptability of the parties to the environment. Exploring these fascinating interactions helps shed light on the complex ecological and evolutionary implications of social relationships in the plants-AM fungi-bacteria continuum. However, the study on reciprocity between AM fungi and bacteria in the hyphosphere mediated by plants has just begun, and we need more experimental evidence to explore the role of different forms of interactions between them in the stable development of microbial communities. The information behind these interactions can be explored from broader interdisciplinary perspectives and the application of cutting-edge technologies. For example, genetics and evolutionary theory can thoroughly investigate the molecular mechanisms, regulatory networks and horizontal gene transfer of plant-AM fungal-bacterial cooperation. Raman microspectroscopy and “transparent soil” microcosms allow direct visualization of the physiological status, phenotypes, and functions of plants, AM fungi and bacteria in invisible solid matrices. In addition, multidimensional and multimodal datasets of microorganisms, reciprocal models or theoretical frameworks, or even predictive tools based on artificial intelligence, can be applied, paving the way for future studies into cooperation between different species along this continuum.

## Data Availability

Data sharing not applicable to this article as no datasets were generated or analyzed in the current review.
